# Effectiveness of a tailored intervention to improve cardiovascular risk management in primary care: study protocol for a randomised controlled trial

**DOI:** 10.1186/1745-6215-14-433

**Published:** 2013-12-17

**Authors:** Elke Huntink, Naomi Heijmans, Michel Wensing, Jan van Lieshout

**Affiliations:** 1 Radboud University Nijmegen Medical Center, Scientific Institute for Quality of Healthcare, Postbus 9101, 6500 HB, Nijmegen, The Netherlands

**Keywords:** Cardiovascular disease, Tailored implementation of chronic diseases, General practices, Practice nurses, Tailored implementation program, E-health, Physical activity, Motivational interviewing

## Abstract

**Background:**

Cardiovascular disease (CVD) is an important worldwide cause of mortality. In The Netherlands, CVD is the leading cause of death for women and the second cause of death for men. Recommendations for diagnosis and treatment of CVD are not well implemented in primary care. In this study, we aim to examine the effectiveness of a tailored implementation program targeted at practice nurses to improve healthcare for patients with (high risk for) CVD.

**Methods/design:**

A two-arm cluster randomized trial is planned. We offer practice nurses a tailored program to improve adherence to six specific recommendations related to blood pressure and cholesterol target values, risk profiling and lifestyle advice. Practice nurses are offered training and feedback on their motivational interviewing technique and an e-learning program on cardiovascular risk management (CVRM). They are also advised to screen for the presence and severity of depressive symptoms in patients. We also advise practice nurses to use selected E-health options (selected websites and Twitter-consult) in patients without symptoms of depression. Patients with mild depressive symptoms are referred to a physical exercise group. We recommend referring patients with major depressive symptoms for assessment and treatment of depressive symptoms if appropriate before starting CVRM. Data from 900 patients at high risk of CVD or with established CVD will be collected in 30 general practices in several geographical areas in The Netherlands. The primary outcome measure is performance of practice nurses in CVRM and reflects application of recommendations for personalized counselling and education of CVRM patients. Patients’ health-related lifestyles (physical exercise, diet and smoking status) will be measured with validated questionnaires and medical record audit will be performed to document estimated CVD risk. Additionally, we will survey and interview participating healthcare professionals for exploration of processes of change. The control practices will provide usual care.

**Discussion:**

Tailored interventions can improve healthcare. An understanding of the methods to reach the improved healthcare can be improved. This research contributes a share of it. Identification of the determinants of practice and developing implementation interventions were two steps which were completed. The subsequent step was implementation of the tailored intervention program.

**Trial registration:**

Name trial register: Nederlands trial register

Web address of trial register: http://www.trialregister.nl

Data of registration: 11 July 2013

Number of registration: NTR4069

## Background

Cardiovascular disease (CVD) is an important cause of mortality and reduced quality of life worldwide [1]. In The Netherlands, CVD is the leading cause of death for women and the second cause of death for men [2] and imposes a heavy burden on both patients and health care, resulting in high expenditures [3]. Studies have found that primary care for cardiovascular risk management (CVRM) is suboptimal for substantial numbers of patients [4,5]. This is partly related to unfavourable lifestyles of many patients, which are difficult for patients to change and difficult for healthcare professionals to manage [6,7]. Patient education and counselling in primary healthcare can moderately improve patients’ lifestyle and self-management [7] but it remains a challenge to implement effective methods of patient education and counselling widely and sustainably in primary care.

The recommendations for diagnosis and treatment of CVD have been summarized in multidisciplinary clinical practice guidelines, including in The Netherlands, which will be the setting of our study [8]. While it includes general recommendations on items of patient education, prevailing clinical guidelines pay little attention to how this is best organised in busy daily practice. In The Netherlands, the latter is provided in related guidelines, called ‘care standard’, which focuses on organisation of cardiovascular risk management [9]. However, both the clinical guidelines and the ‘care standard’ do not provide detailed guidance for how to implement this in daily practice [6]. A challenge therefore remains in encouraging patient self-management, informing patients, guiding patients towards a healthy lifestyle and cooperation between healthcare professionals.

Firstly, to enhance the current care, six key recommendations were selected from the Dutch multidisciplinary guideline for CVRM (Table 1).

**Table 1:** Recommendations for cardiovascular risk management (CVRM)

	**Recommendations**
1.	Systolic blood pressure (SBP) < 140 mmHg in patients at high risk for CVD
2.	Systolic blood pressure < 140 mmHg in patients with established CVD
3.	Low density lipoprotein (LDL) cholesterol < 2.5 mmol/l in patients at high risk for CVD
4.	Low density lipoprotein (LDL) cholesterol < 2.5 mmol/l in patients with established CVD
5.	Promote lifestyle changes in patients with (high risk for) CVD
6.	Create a risk profile for patients with chronic kidney disease

Subsequently, 11 determinants of practice were selected. The identified determinants were categorized under four headings; (1) healthcare professional related factors, (2) patient related factors, (3) professional interaction and (4) incentives and recourses. The first heading ‘health professional-related factors’ included four determinants: (1) clinical inertia, (2) encouragement of general practitioners and practice nurses to apply motivational interviewing more often, (3) provision of patients with good advice and explanations, and (4) more attention is needed for patient motivation. The second heading ‘patient related factors’ also included four determinants: (1) patients should be encouraged to adopt and implement lifestyle advice provided by general practitioners and practice nurses, (2) patients should be able to ask for more information, (3) feasible targets for the patient should be drafted, and (4) more attention for patient compliance is needed. Under the third heading ‘professional interaction’, the single identified determinant here stated that communication should be improved between healthcare professionals in primary and secondary care. The last heading consists of ‘incentives and resources’ for which two determinants were found: (1) self-management should be promoted by using E-health, and (2) practice nurses and protocols should be available in general practice. These determinants were the basis for the implementation program.

This study is part of the Tailored Implementation for Chronic Diseases (TICD) project, which has the overall aim to develop and test methods of tailoring implementation interventions to determinants of practice for knowledge in chronic illness care [10].

### Aims and research questions

The primary aim of the study is to examine the effectiveness of a tailored implementation intervention for improving the professional performance of practice nurses for patients at high risk for CVD or established CVD in primary care. The secondary aim is to examine the validity of the process of tailoring implementation interventions to determinants of practice.

### Research questions

1. What is the effectiveness of a tailored implementation program compared to usual care on the professional performance of practice nurses and patient related outcomes?

2. What is the validity of the methods used to tailor the implementation program to determinants of practice?

## Methods/design

### Trial design

**Figure 1:**
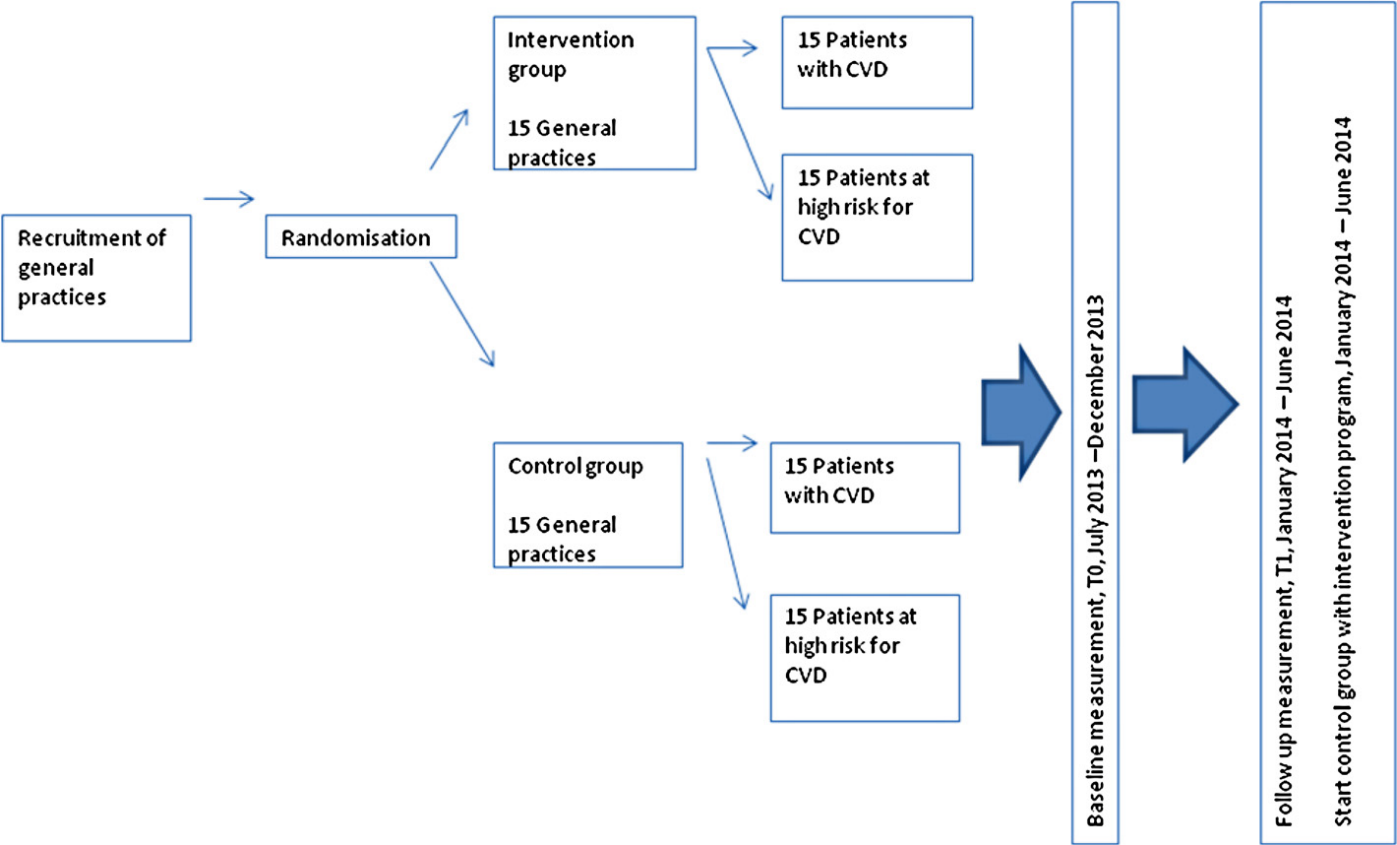
**Study flow chart.** This table provides a time schedule in which timeframe the intervention is conducted.

This study is a two-arm cluster randomised trial to determine the effectiveness of a tailored intervention program targeted at practice nurses in primary care and patients at risk for CVD or with established CVD. We will include practice nurses and patients from primary care practices in The Netherlands. The general practices are randomised into two study arms; (1) the intervention group, in which practice nurses and patients are offered a tailored intervention program, and (2) the control group in which practice nurses provide usual care and patients are not offered any interventions while the intervention program is implemented.Usual care consists of consults in which practice nurses provide lifestyle advices on diet, exercise, smoking, and alcohol use. General practitioners are responsible for medication prescriptions. Rates of consults vary from various contacts within weeks during medication dose adjustments to once a year, depending on actual values of treatment parameters and patients’ preferences. After the project period in the intervention group, the intervention program is offered to the control group (see Figure 1).

### Ethical approval

This study was approved by the Medical Ethics Committee of CMO region Arnhem - Nijmegen; the study is registered as 2013/229.

### Setting

#### General practices

A random sample of general practices in several geographical areas in The Netherlands will be invited to participate in the study. After being given approval (by the general practitioner or the practice nurse), general practices will be randomly allocated to the intervention program or control group. All interventions and data collection procedures are planned for between July 2013 and June 2014.

### Participants

#### Practice nurses

The implementation program is primarily targeted at practice nurses. Practice nurses eligible for inclusion in the study have CVRM as their task and have been trained for motivational interviewing during their education or as an additional training. Practice nurses will perform measurements of patients’ biomedical parameters and provide them with lifestyle advice and also consult the general practitioners about medication policy.

#### Patients

Eligible patients’ will be extracted from the medical records by using International Classification of Primary Care (ICPC) codes, K74-K76, K85-K92, K99.1 and T93. Eligible patients are adults aged 18 or older, have a high risk of CVD (but no known CVD) or established CVD and are capable of providing informed consent. These high risk patients have a risk score of 20% or higher for morbidity and mortality due to CVD based on age, gender, smoking status, systolic blood pressure and total cholesterol/high density lipoprotein (HDL) cholesterol ratio [8]. Exclusion criteria are: (1) diabetes mellitus, (2) pregnancy and lactation, (3) terminal illness, (4) cognitive impairment, and (5) poor language skills. Patients with diabetes mellitus will be excluded because this illness has its own guidelines/standard of care. Diabetes care is well developed and monitoring CVD patients with diabetes as co-morbidity would mainly evaluate diabetes care.

A random sample of patients who meet the criteria will be invited by a letter, which provides comprehensive information about the intervention program. Contact details of TICD researchers are provided so that patients can ask for additional information if desired. Patients will return their informed consent with permission for audit of their medical records during the trial, to Radboud University Nijmegen Medical Center in a postage-paid envelope.

### Development of the implementation program

**Figure 2:**
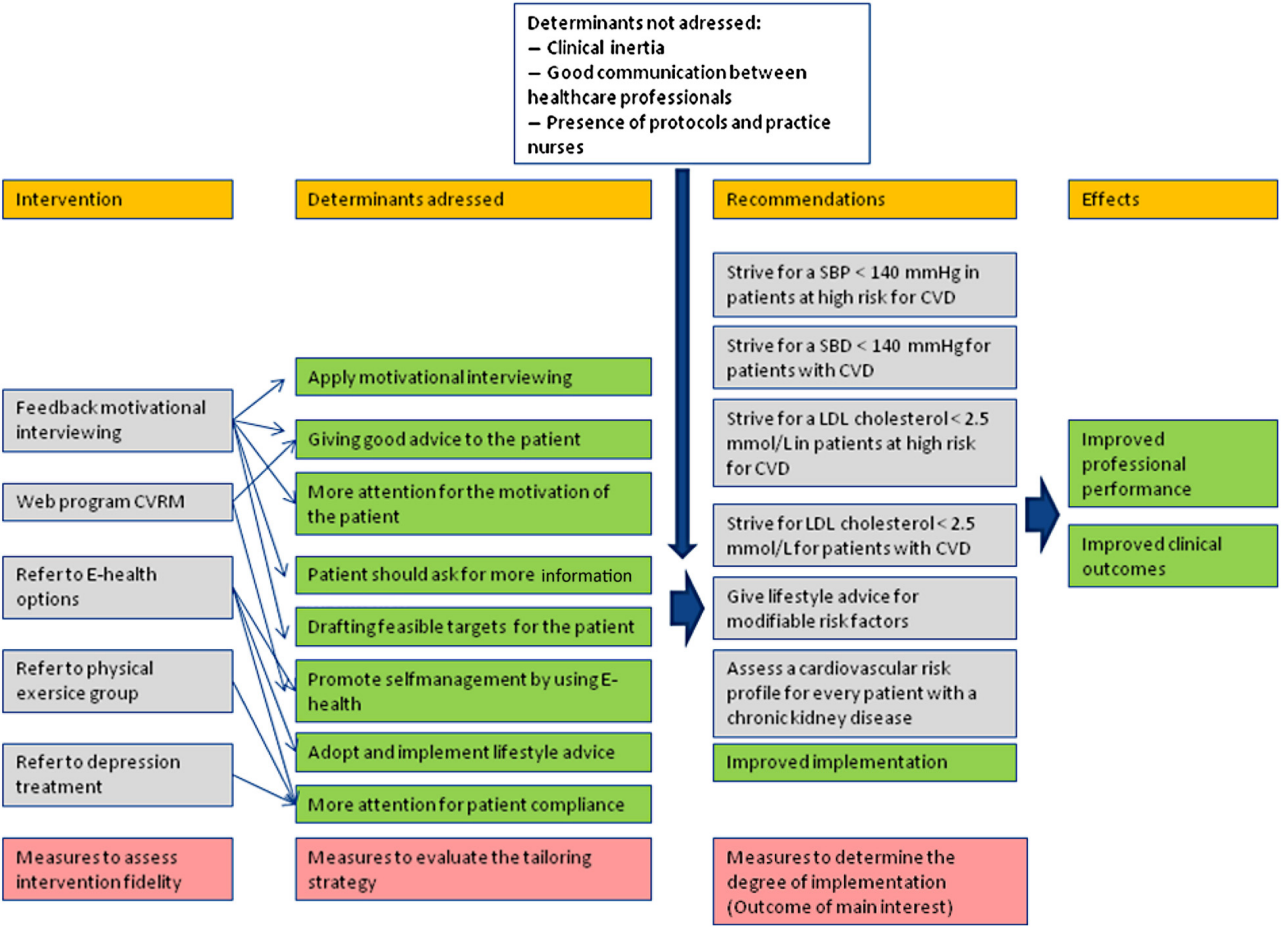
**Logic model.** This table provides information regarding which determinants and recommendations are addressed to the intervention program and which are not addressed, as well as showing the intended effect.

In previous phases of the TICD project, determinants for the implementation of the aforementioned six recommendations have been identified as well as strategies for addressing those determinants. This process is reported in detail elsewhere [10]. On the basis of this prior work, a tailored implementation intervention has been developed, in which each strategy addresses one or more specific determinants, see the logic model, Figure 2.

### Implementation program

The implementation program is based on extensive research on determinants of practices and potential implementation interventions, which can address relevant determinants. Some adaptation (further tailoring) at the level of practice nurses and patients is planned by a structured procedure for translating the treatment protocol for enhanced counselling for local use. In particular, concrete options for E-health and physical exercise in a local community will be specified by the practice nurse on a written treatment protocol. The following implementation interventions are offered:

#### Refresher training on motivational interviewing and CVRM-knowledge

Practice nurses are closely involved in patient care and capable of performing substantial parts of the standard of care CVRM [8]. To further enhance their knowledge and skills, two refresher trainings will be offered; one on motivational interviewing and one on knowledge of CVRM.

##### Motivational interviewing

Practice nurses will be guided by a professional trainer who is affiliated with MINTned (Dutch Association of Trainers in Motivational Interviewing). The trainer will provide feedback directly after two consecutive consultations by using the Behaviour Change Counselling Index (BECCI) [11] code. During this procedure, the practice nurse can directly apply feedback and feel confident that they have applied it appropriately. Previous research has shown that providing feedback is effective for improving motivational interviewing techniques among nurses and that there is room for improvement [12-15].

##### CVRM-knowledge

For enhancing knowledge of CVRM, we recommend a recently launched e-learning program. This program is specifically designed for practice nurses by the Dutch College of General Practitioners and consists of several modules with information about tasks areas of practice nurses involved in CVRM. At the end of every module, the practice nurse is required to answer several questions.

##### Instruction in E-health and application of Twitter-consult

A short instruction will be offered which emphasizes advantages of using E-health in primary care and describes how this medium can be used effectively by patients. During this instruction, we will recommend the following websites which are selected after careful exploration of available options: ‘thuisarts.nl’ (‘general practitioner at home’, developed by the Dutch College of General Practitioners), and ‘hartenvaatgroep.nl’ (‘heart and vessel group’) which contain carefully selected and reliable information on health and disease for both patients and the general public. These websites are easy to use by patients and searches can be completed by using search terms or by clicking organs on a picture of a human body. Practice nurses will discuss with the patients the opportunities to access the Internet.

In addition to using informative websites, practice nurses are asked to notify patients on a Twitter-consult. In this Twitter-consult, a general practitioner will answer questions about CVRM.

### Clinical interventions in the implementation program

We aim to target the six chosen treatment recommendations by enhancing the tailored implementation program of improved counselling by practice nurses which will improve patient self-management. These are provided in prevailing clinical guidelines for CVRM [8], on which we elaborated a few additional procedures. As patients with CVD have a higher risk for experiencing depressive symptoms [16-19] which can seriously impair the ability of patients to change their lifestyle habits [20], we will suggest that practice nurses pay particular attention, and plan action, according to the presence of depressive symptoms. When the practice nurse has doubts about the presence of depressive symptoms in the patient, the Patients Health Questionnaire (PHQ-9) can be used as supportive material.

This is consistent with guideline recommendations, which state that counselling needs to be tailored to individual patients’ capabilities. The following approach will be recommended:

• No depressive symptoms

Patients without symptoms of depression are considered to be eligible for a more independent approach in changing and managing their health behaviours. Therefore, they will be referred to several E-health options. These will consist of educational websites, as mentioned in the E-health training for practice nurses, on which patients can search for information suited for individual goals in CVRM and the Twitter-consult. Although the latter is not particularly emphasised in prevailing guidelines, research found that Internet interventions can reduce cardiovascular risk [21-23] and reduce the number of visits to healthcare providers [24].

Furthermore, and for providing additional support, patients will be given a card on which websites, dates of the Twitter-consult, and target values for blood pressure and cholesterol are stated. This card will be the size of a credit card so that it is easy for patients to keep it with them and use it as a reminder for treatment targets and sources of information to reach their treatment goals.

• Mild depressive symptoms

Practice nurses are recommended to refer patients with mild symptoms of depression to a physical exercise group. A physical exercise group can be particularly suited for these patients as it combines social support and physical exercise, both of which have a beneficial effect on cardiovascular health and on depressive symptoms [25-27]. The specific form of this exercise group will depend on what is available in the local community in which the practice is situated. Examples are exercise groups led by physical therapists or exercise groups at the local gym.

• Major depressive symptoms

In patients with major depressive symptoms, practice nurses are recommended to refer these patients as appropriate within their practice and not to start CVRM until relief of depressive symptoms has been achieved.

### Control group

In the control group, no intervention is provided to practice nurses. Patients will receive usual care.

After the project period in the intervention group, the intervention program is offered to the control group.

After analyzing all data, results will be provided to all the general practices in the intervention and control groups.

## Outcomes and measurements

### Outcomes

#### Primary outcome

The primary outcome refers to the professional performance of practice nurses and reflects application of recommendations for personalised counselling and education of CVRM patients. As a primary outcome, a dichotomous score is created for measurement in each patient, reflecting adequate or inadequate performance. We considered practice nurses’ professional performance to be adequate when at least one of the following conditions is met:

1) There is a record in the patient’s medical file, or other healthcare provider-based records, that the patient has received advice on at least one lifestyle item as specified in prevailing guidelines of CVRM; diet, smoking or physical exercise, and which are relevant for the individual patient in the previous six months. Also, at least one target for improving an aspect of lifestyle is recorded. This target is maximized 15 months previously. When a patient has a perfect lifestyle then that will be recorded.

2) There is a notation in the patient’s medical file that the patient has none, mild or major depressive symptoms and that the patient has been referred to E-health, a physical exercise group, or depression treatment respectively.

#### Secondary outcomes

The secondary outcomes consist of the following:

• Practice nurses

Quality of effective referral

Using data from patients’ medical files and the patient questionnaires, we will assess whether practice nurses referred patients to treatment options (E-health, a physical exercise group, or depression treatment) in accordance with our recommendations on depressive symptoms. This measurement of quality represents correct referral and is thus an extension of measurement of referral as defined in the primary outcome.

Quality of motivational interviewing

For assessment of quality of motivational interviewing skills, audio-taped interviews of practice nurses in the intervention group and control group will be transcribed verbatim and will be coded by trainers using the Motivational Interviewing Treatment Integrity (MITI) code. Results of these codings will be compared with baseline after six months.

• Patients

Cardiovascular risk predictors

Using prevailing risk estimation tables (based on the Euro score data), the following parameters are used for calculation of the risk score for patients at high risk for CVD; age, gender, smoking status, systolic blood pressure and total cholesterol/HDL- cholesterol ratio). For patients with established CVD smoking status, systolic blood pressure and total cholesterol/HDL-cholesterol ratio will be used. Change of the parameters will be measured before and after implementation of the tailored intervention.

Self-management

Using a composite questionnaire, we will assess whether patients applied lifestyle advice for improving their self-management. This questionnaire will be sent at the start of the implementation program and after six months. The questionnaire will be sent to the patient’s home address.

### Measurement procedures

In each general practice, measurements on practice nurses and patients are performed at baseline and at follow-up after six months.

The following measurement methods will be used: medical record audit, patient questionnaires, questionnaires for practice nurses and the MITI code for assessing motivational interviewing skills. Specific measures will include:

1. Indicators for clinical performance, using a modified version of a validated abstraction tool for a medical audit in patients at moderate-high risk for, and with, established CVD from the EPA Cardio instrument. Additional information about medication and other chronic diseases will be measured as well. These data will be collected from medical records [3].

2. Health related lifestyles. Questionnaires for specific aspects of lifestyle of patients will be used, including physical exercise (Rapid Assessment of Physical Activity (RAPA), 9 items) [28], diet (reduced Rapid Eating and Activity Assessment (REAP-S), 12 items) [29] and smoking behaviour [30].

3. Other measures on patients, include items on demographic characteristics, healthcare use, changes in patient activation (Patient Activation Measure (PAM), 13 items) [31], Report on adherence to medication (Medication Adherence Measure, 4 items) [32], the Patient Assessment of Chronic Illness Care (PACIC, 26 items) [33]. The depression related items in the Patient Health Questionnaire list will be used for measurement of depressive symptoms (PHQ-9, 9 items) [34]. Data of quality of life will be collected using the EQ-5D (6-items plus Visual Analogue Scale (VAS) [35].

4. A questionnaire for practice nurses will be provided, containing items on demographic characteristics, general practice characteristics, education, familiarity with motivational interviewing and years of employment as practice nurse. The questionnaire will also include questions about participation in the offered e-learning program.

5. For assessment of motivational interviewing skills, we will use the Motivational Interviewing Treatment Integrity code [36] for the transcribed interviews. The assessor will be blinded for intervention or control group and for the first or second consult.

All the completed questionnaires will be sent to Radboud University Nijmegen Medical Center in a postage-paid envelope. The questionnaires are marked with a unique number, and will be stored in a locked closet.

#### Process evaluation

Following the international study protocol for the TICD project [37], the aim of the process evaluation is two-fold: to examine the fidelity of the planned intervention strategy and how this relates to the effectiveness of the implementation program, and to identify possible mechanisms underlying effectiveness (or lack of it) on primary and secondary outcomes.

As a complement to the international protocol, interviews will be held with randomly selected patients who participated in this research. Data about the professional performance of the practice nurse, social support and using offered E-health options will be collected. During this interview, the following determinants as perceived by the patients will be evaluated: provision of good explanation for patients; patients’ need for knowledge; whether enough attention is payed to patient motivation and using E-health options for promoting self-management.

The MITI code measures the extent to which the practice nurse uses empathic statements. The use of empathic statements will be investigated more extensively using the Empathy Quotient questionnaire [38].

### Sample size

The study is powered to detect a 15% difference (17 to 32%) in provided lifestyle advice on all lifestyle variables included in the risk score and as recorded in patients’ medical files [30]. The sample size calculation assumed an intra-cluster correlation coefficient (ICC) of 0.05, alpha of 0.05, and a power of 0.80 and indicated that 450 patients per group will be needed (15 patients at high risk for CVD and 15 patients with established CVD per cluster, sampled in 30 practices).

### Recruitment

The aim is to include 30 general practices. Addresses of 1,600 general practices will be obtained from a national database. We will start by sending 800 invitations. Postal reminders will be sent to non responders after two weeks. When insufficient general practices are recruited, another 800 invitations will be sent, together with a reminder to non responders after two weeks.

Per general practice, 15 patients with established CVD and 15 patients at high risk for CVD will be included. Assuming a response rate of 33% of the patients at high risk for CVD and a drop out rate of 35%, we will invite 69 patients at risk for CVD per practice. Similarly, taking into account a response rate of 50% for patients with established CVD and a drop out rate of 35%, 46 patients with established CVD will be invited per general practice.

### Randomisation

Randomisation of general practices will be done by an independent research assistant through a computer. The general practices will be allocated randomly to two equal sized groups for the intervention program and a control group. Block randomisation for practice size and rural/urban area will be performed to control for differences in work processes within small and large general practices

### Blinding

Due to the nature of the intervention program, blinding of the patients and practice nurses will not be possible for this intervention program.

### Data collection methods

Data collection methods are described in the section of outcomes.

### Statistical methods

Data will be analyzed using SPSS (version 20, IBM Corp.) and SAS (version 9.2, SAS Institute Inc.) For all statistical testing, two-sided hypothesis testing with an alpha level of 0.05 will be applied. Baseline descriptions and comparisons of practices, practice nurses, and patients will be provided (percentages or means and standard deviations where appropriate) using *X*^2^ tests for categorical data and *t*-tests for continuous data. All data analyses will be based on ‘intention to treat’.

For assessing differences in the primary outcome (professional performance of practice nurses), the intervention and control group will be compared in terms of provision of advice on all lifestyle variables and determination of at least one lifestyle target for improvement using a X^2^ test.

An additional analysis of the primary outcome will be performed by applying a logistic regression analysis which uses professional performance as dependent variable and group allocation, practice nurse characteristics, refresher training (on motivational interviewing and CVRM), and referring patients to the different options as independent variables.

For the secondary outcomes, ‘quality of effective allocation’ and ‘quality of motivational interviewing’, a X^2^ test and *t*-test will be performed respectively.

For assessment of the secondary outcomes reduction in risk score and enhancement of self-management in the two groups, we plan to perform multilevel regression analyses which use the risk score and a composite score for self-management at six months as dependent variables. Independent variables will include, diverse patient characteristics (age, sex, social economic status (SES), co-morbidity), depressive symptoms (none, mild, or major), group allocation (E-health, physical exercise group, depression treatment), quality of effective referring patients, lifestyle advice on treatment parameters and lifestyle targets.

## Discussion

This implementation program has been developed with the aid of the strategy tailored implementation interventions. This strategy exists of three key steps: identification of the determinants of healthcare practice, designing implementation interventions appropriate to the determinants, and application and assessment of implementation interventions that are tailored to the indentified determinants. In order to achieve desired changes in healthcare practice in particular the healthcare for patients with established CVD or at high risk in The Netherlands [10,39]. This research focuses on the implementation of the intervention program and the final evaluation.

The results of this trial will be directly applicable to primary care settings. Should the interventions delivered at the level of the practice nurses found to be effective in improving patients’ quality of life and lifestyle, then the findings would be accessible for wider application.

## Trial status

Invitations are sent to the general practitioners. The first general practices are visited. The practice nurses in the intervention group and control group did received explanation about this research and what is expected from them.

## Abbreviations

BECCI: Behaviour Change Counselling Index; CVD: Cardiovascular disease; CVRM: Cardiovascular risk management; HDL: High density lipoprotein; ICPC: International Classification of Primary Care; LDL: Low density lipoprotein; MITI: Motivational interviewing treatment integrity; PACIC: Patient assessment of chronic illness care; PAM: Patient activation measure; PCE: Personalized counselling and education; PHQ-9: Patient health questionnaire; RAPA: Rapid assessment of physical activity; REAP-S: Rapid eating and activity assessment; SBP: Systolic blood pressure; SES: Social economic status; TICD: Tailored implementation for chronic diseases; VAS: Visual analogue scale.

## Competing interests

The authors declare that they have no competing interests.

## Authors’ contributions

EH wrote the draft version of this protocol, which was commented on by NH, JvL and MW. MW is the project leader of TICD project. All authors critically assessed and approved this study protocol. This study protocol has been discussed with and reviewed by the project partners.
